# Efficacy of pyrethroid-pyriproxyfen and pyrethroid-chlorfenapyr nets on entomological indicators of malaria transmission: third year of a randomised controlled trial in Benin

**DOI:** 10.1038/s41598-024-63883-2

**Published:** 2024-06-05

**Authors:** Arthur Sovi, Boulais Yovogan, Constantin J. Adoha, Bruno Akinro, Manfred Accrombessi, Edouard Dangbénon, Landry Assongba, Albert Sourou Salako, Germain Gil Padonou, Louisa A. Messenger, Corine Ngufor, Jackie Cook, Natacha Protopopoff, Martin C. Akogbéto

**Affiliations:** 1 Faculté d’Agronomie, Université de Parakou, Parakou, Benin; 2 Centre de Recherche Entomologique de Cotonou, Cotonou, Benin; 3 Faculty of Infectious and Tropical Diseases, Department of Disease Control, The London School of Hygiene and Tropical Medicine, London, UK; 4 Faculté des Sciences et Techniques, Université d’Abomey-Calavi, Abomey-Calavi, Benin; 5 Parasitology and Vector Biology Laboratory (UNLV PARAVEC Lab), School of Public Health, University of Nevada, Las Vegas, NV USA; 6 Department of Environmental and Occupational Health, School of Public Health, University of Nevada, Las Vegas, NV 89154 USA; 7 Medical Research Council (MRC) International Statistics and Epidemiology, Epidemiology Group, London School of Hygiene and Tropical Medicine, London, UK

**Keywords:** Zoology, Diseases

## Abstract

The present cluster-randomised control trial aims to assess the entomological efficacy of pyrethroid-pyriproxyfen and pyrethroid-chlorfenapyr LLINs compared to the standard pyrethroid-only LLINs, in their third year of community usage. Adult mosquito collections were performed every 3 months, in 4 randomly selected houses in each of the 60 trial clusters, using human landing catches. Adult mosquitoes were morphologically identified and *Anopheles* vectors were molecularly speciated and screened for the presence of the L1014F *kdr* mutation using PCR. *Plasmodium falciparum* sporozoite infection was assessed using ELISA. A subset of *An. gambiae* s.l. was also dissected to examine parity and fertility rates across study arms. There was no evidence of a significant reduction in indoor vector density and entomological inoculation rate by the pyrethroid-pyriproxyfen [DR 0.94 (95% CI 0.46–1.88), p = 0.8527; and RR 1.10 (95% CI 0.44–2.72), p = 0.8380], and pyrethroid-chlorfenapyr [DR 0.74 (95% CI 0.37–1.48), p = 0.3946; and RR 1.00 (95% CI 0.40–2.50), p = 0.9957] LLINs, respectively. The same trend was observed outdoors. Frequencies of the L1014F *kdr* mutation, as well as parous and fertility rates, were similar between study arms. In the third year after net distribution, entomological indicators show that the two dual active-ingredients nets performed similarly to the standard pyrethroid-only LLIN. To maintain malaria gains, it is crucial that net distribution cycles fit with their operational lifespan.

## Introduction

Long-lasting insecticidal nets (LLINs) contributed to preventing the occurrence of approximately 1.5 billion malaria cases and 7.6 million malaria deaths over the two past decades^1^. However, there was a stagnation in the number of global malaria cases between 2015 and 2018^2^, followed by a resurgence in disease transmission from 2019, particularly in some sub-Saharan Africa settings^3^. One possible reason for this may be widespread pyrethroid insecticide resistance in malaria-transmitting vectors^4^. There is an urgent need for novel types of LLINs able to provide improved disease control in a context of transmission rebound. A new generation of LLINs developed over the past few years incorporate a pyrethroid plus a partner insecticide with a different mode of action, such as pyriproxyfen, a growth regulator which sterilizes mosquitoes, or chlorfenapyr, which affects the mitochondria and disrupts the production of cellular energy required for flight.

The findings of the two first years of the present cluster-randomized controlled trial (RCT) demonstrated that the pyrethroid-chlorfenapyr LLIN significantly reduced vector density and the entomological inoculation rate (EIR) by 56% and 66%, 53% and 70%, in the first and second year of the trial, respectively. However, the pyrethroid-pyriproxyfen LLIN significantly reduced the EIR by 58% only during the first year of the trial, but did not affect either of the two indicators (vector density, and EIR) in the second year of the trial^5^. Similarly, in participants of any age, malaria infection prevalence was significantly reduced by 40% by the pyrethroid-chlorfenapyr LLIN at 18 months after LLIN distribution; a significant reduction in malaria prevalence was not observed with the pyrethroid-pyriproxyfen LLIN^5^. The same trend was observed in another RCT conducted in Tanzania^6^, which led the World Health Organization (WHO) to grant a full recommendation for the deployment of pyrethroid-chlorfenapyr LLINs in pyrethroid resistance areas, and a conditional recommendation for the use of pyrethroid-pyriproxyfen LLINs^7^.

Typically, net replacement in the community is conducted every three years through mass distributions led by National Malaria Control Programmes (NMCP). Based on previous data of field performance of standard pyrethroid-only, LLINs are expected to remain biologically efficacious over 3 years of community usage^8^. However, recent field studies indicate that net bio-efficacy and durability can vary substantially between net types and in different communities^9^. In the third year of the RCT conducted in Tanzania, the pyrethroid-chlorfenapyr LLIN was found to significantly reduce the vector density by 54% and the EIR by 68% compared to the pyrethroid-only LLIN while the pyrethroid-pyriproxyfen LLIN did not^10^. Similarly, malaria infection prevalence was significantly reduced by 43% with the pyrethroid-chlorfenapyr LLIN, while there was no evidence of a reduction of this indicator with the pyrethroid-pyriproxyfen LLIN^10^.

The present study reports the impact of pyrethroid-pyriproxyfen and pyrethroid-chlorfenapyr LLINs on entomological indicators in the third year of a RCT conducted in an area of high pyrethroid resistance in Benin.

## Methods

### Study area

The study was a three-arm, parallel, RCT that took place in Covè (07°13′08.0400″N, 02°20′21.8400″E), Ouinhi (07°05′00″N, 02°29′00″E), and Zagnanado (07°16′00″N, 02°21′00″E), three districts of the Zou department located 154 km away from Cotonou, the economic capital of Benin. The area is characterized by intense malaria transmission driven by *An. coluzzii* and *An. gambiae* s.s. populations, which are both highly resistant to pyrethroid insecticides, driven by several resistance mechanisms, including the L1014F *kdr* mutation and overexpression of mixed function oxidases (MFOs)^11^.

**Figure 1:**
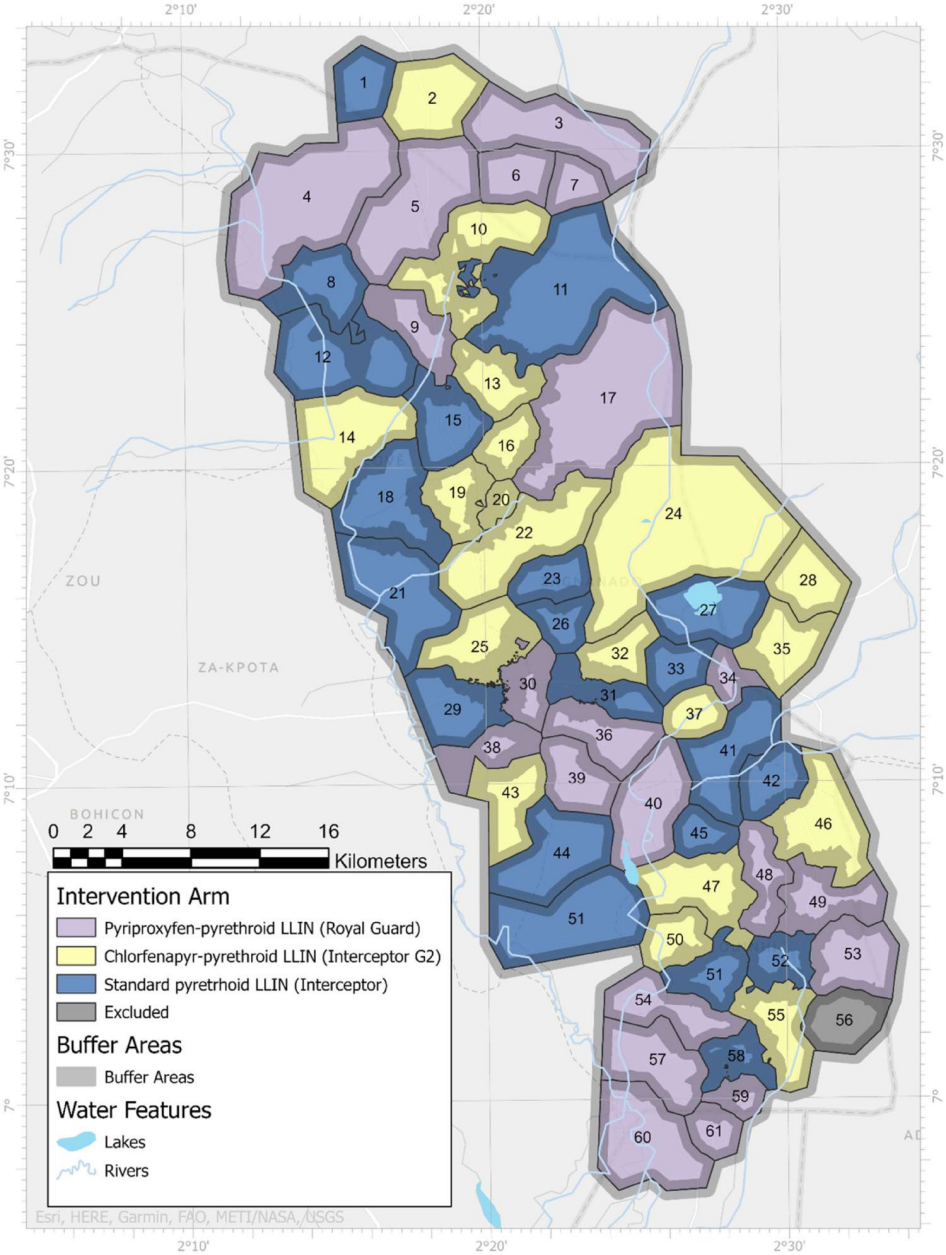
Map showing the 20 clusters of each of the three study arms. The map was drawn by study investigators (M.A., E.D., J.C.) using Esri ArcGIS Pro 3.1 software (https://pro.arcgis.com/fr/pro-app/latest/get-started/download-arcgis-pro.htm), study and online data provided by GADM (https://gadm.org/download_country.html), and Natural Earth (https://www.naturalearthdata.com/).

The 123 villages of the study area were divided into 60 clusters, with 20 randomly allocated to each of the three followings arms^5^: (i) pyrethroid-chlorfenapyr LLIN: Interceptor® G2 (Intervention 1); (ii) pyrethroid-pyriproxyfen LLIN: Royal Guard® (Intervention 2); and (iii) standard pyrethroid-only LLIN: Interceptor® (Control arm) (Fig. 1). Each cluster had a core area, and a buffer area of at least 1000 m for a minimum of 100 households. Entomological indicators were only measured in the core area, although the interventions were deployed in the whole cluster.

### Mosquito collection and processing

#### Human landing catches (HLCs)

The present study took place between June 2022 and April 2023, with collections performed in all 60 clusters every three months, equating to a total of four collection rounds over the study period. In each cluster, 4 houses per round (1 randomly selected, and 3 others chosen by the field team, 15–20 m around the first one) were sampled from 07:00 p.m. to 06:00 a.m. using human landing catches (HLCs). In each house, two volunteers (one sitting indoors, and the second outdoors) collected all mosquitoes that landed on their lower limbs using haemolysis tubes and flashlights. For the entire study period, a total of 960 collections (4 indoor collectors × 60 clusters × 4 rounds) were performed indoors. The same number of collections were also performed outdoors.

Mosquitoes collected through HLCs were morphologically identified to species-level using the taxonomic keys of Gillies and Meillon^12^. Heads-thoraces of a subset (32.4%: 9356/28,915) of unfed specimens of *An. gambiae* s.l., randomly selected indoors and outdoors, and across collection hours in each cluster, were screened by ELISA-CSP to detect *P. falciparum* sporozoite infection^13^, and dissected to determine the parous rate^14^. Legs and wings of the same samples were used for molecular identification of sibling species^15^ and presence/absence of the L1014F *kdr* mutation^16^.

#### Collection using mouth aspirators

**Figure 2:**
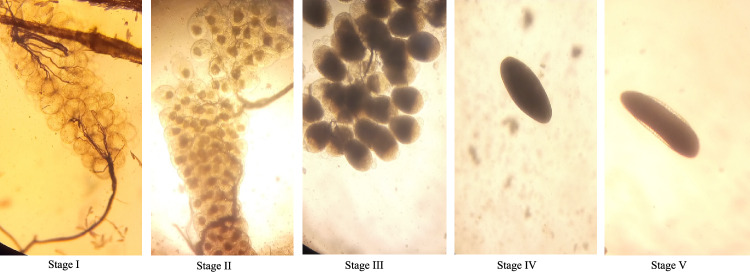
Christopher’s egg stages (I–V). The eggs was visualized under microscope at 10 × magnification. The image was produced by study investigators (A.S.O., B.Y., and C.J.A.).

To assess the impact of pyriproxyfen on the fertility of *An. gambiae* s.l., four clusters in each of the three study arms were surveyed over two rounds of collection (August 2022 & January 2023). At each round of collection, 10 houses randomly chosen per cluster were sampled between 07:00 to 09:00 a.m., by two entomology technicians and two local guides. Thus, a total of 240 houses (10 houses × 4 clusters × 3 arms × 2 rounds) were surveyed. In each house, all mosquitoes that rested on hung clothes, walls, furniture, roofs, earthen jars and others were collected using mouth aspirators. Thereafter, they were released into paper cups, provided with a 10% sweetened sugar solution, placed in cooler boxes, and carried to the field-laboratory where blood-fed *An. gambiae* s.l. were morphologically identified and released in cages for a three-day-resting time which allowed for blood-meal digestion, prior to their dissection and determination of Christopher’s egg stage (I–V) of development^17^ (Fig. 2). Of note, the dissection was performed by delicately pulling the last two abdominal segments of each mosquito, which allowed to extract the ovaries. A mosquito was considered fertile when it had stage V eggs.

### Data analysis

For both locations (indoor and outdoor), the nightly vector density was calculated at the household level by dividing the total number of *Anopheles* vectors (*An. gambiae* s.l. + *An. funetus* s.l. + *An. nili* s.l.) collected by the total number of collection nights for each round. The number of sporozoite positive vectors was divided by the total number of tested vectors to determine the sporozoite rate (SR). The indoor/outdoor nightly EIR was generated at the cluster level by multiplying the mean indoor/outdoor vector density by the indoor/outdoor SR. The parous rate in *An. gambiae* s.l. was calculated at the cluster level by dividing the number of parous mosquitoes by the total dissected. Similarly, the fertility rate in *An. gambiae* s.l. was estimated at the cluster level by dividing the number of fertile mosquitoes by the total dissected. The resistance mutation frequencies were calculated at the cluster level using the following formula: F = (2 × nRR + nRS)/(2 × (nRR + nRS + nSS)), n = number of a given genotype. Resistance mutation frequencies were analyzed using the exact binomial test.

Vector density and EIR were compared between study arms using a mixed-effect generalised linear model with a negative binomial distribution and with cluster as a random effect. To analyse SR, parous rate, and fertility rate between study arms, a mixed-effect logistic regression was used, with cluster as a random effect. All analyses were performed using Stata 15.0 (Stata Corp., College Station, TX).

### Ethical considerations

Ethical clearance for the present trial was issued by Benin’s National Ethics Committee for Health Research (N°30/MS/DC/SGM/DRFMT/CNERS/SA, Approval n°6 of 04/03/2019), and the Ethics Committee of the London School of Hygiene and Tropical Medicine (16237-1). Prior to their involvement, all mosquito collectors, heads of households, and community leaders gave their informed consent. Only collectors trained to capture mosquitoes prior to being bitten, were involved in the study. Before the start of the trial, they were vaccinated against yellow fever. Whenever they experienced symptoms similar to those of malaria, they were taken care of at the closest health facility. All study procedures were conducted following the relevant guidelines and regulations.

## Results

### Mosquito species composition

Over the third year of the RCT, a total of 106,114 mosquitoes were collected, with the majority (54.3%) outdoors. Overall, *Anopheles* mosquitoes accounted for 35.5% (17,222/48,452) of the indoor collection and 23.8% (13,719/57,662) of the outdoor collection, with a mean of 29.2% (30,941/106,116) of the total (indoor + outdoor) collection. At the arm level, the proportions of *Anopheles* mosquitoes were 28.9% (8831/30,549), 27.7% (10,146/36,671), and 30.8% (11,964/38,894) in the pyrethroid-chlorfenapyr LLIN arm, the pyrethroid-pyriproxyfen LLIN arm, and the standard pyrethroid-only LLIN arm, respectively. Of all collected *Anopheles* mosquitoes, *An. gambiae* s.l. was found in vast majority (92.3%: 28,571/30,941), with peak density observed late at night between 03:00 a.m. and 05:00 a.m. both indoors and outdoors (Supplemental file, Fig. S1). Other *Anopheles* mosquitoes found in lower proportions include *An. funestus* gr, *An. nili* gr, *An. pharoensis*, and *An. ziemanni.*

Of the 4072 specimens of *An. gambiae* s.l. molecularly identified, 70.7% (n = 2880) were *An. coluzzii*, 28.9% (n = 1175) were *An. gambiae* s.s., while the rest was hybrids (*An. gambiae s.s./coluzzii*).

**Figure 3:**
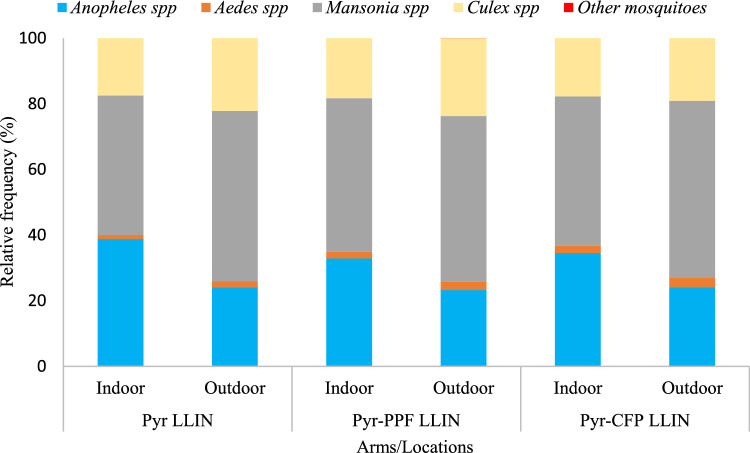
Mosquito species composition in the study area. Pyr LLIN: standard pyrethroid-only LLIN, Pyr-PPF LLIN: pyrethroid-pyriproxyfen LLIN, Pyr-CFP LLIN: pyrethroid-chlorfenapyr LLIN.

In the study area, *Mansonia* spp. was the most collected mosquito both indoors and outdoors in all study arms as it accounted for half of all collected mosquitoes, while *Culex* spp. accounted for one-fifth. *Aedes* spp (2.1%) and other mosquitoes (0.003%) were also found, but at very low frequencies (Fig. 3).

### *Anopheles* vector density, SR and EIR

Indoors, there was no strong evidence of a significant reduction in vector density in the pyrethroid-pyriproxyfen LLIN arm [density = 16.4 bites/person.night (b/p/n), DR 0.94 (95% CI 0.46–1.88), p = 0.8527] and the pyrethroid-chlorfenapyr LLIN arm [density = 14.5 b/p/n, DR 0.74 (95% CI 0.37–1.48), p = 0.3946], as compared to the standard pyrethroid-only LLIN arm (density = 20.4 b/p/n). The same trend was observed outdoors (Table 1).

**Table 1:** *Anopheles* vector density, SR, and EIR.

Locations	Arms	Density					SR			EIR		
		N of *An*	N of collector night	Mean (SD)	DR (95% CI)	p-value	n/N (%, 95%CI)	OR (95% CI)	p-value	Mean (SD)	RR (95% CI)	p-value
Indoor	Pyr LLIN	6537	320	20.4 (32.2)	1 (Ref)		19/2130 (0.9, 0.4–1.8)	1 (Ref)		0.25 (0.75)	1 (Ref)	
	Pyr-PPF LLIN	5234	320	16.4 (22.3)	0.94 (0.46–1.88)	0.8527	22/1548 (1.4, 0.9–2.3)	1.34 (0.65–2.77)	0.4300	0.24 (0.58)	1.10 (0.44–2.72)	0.8380
	Pyr-CFP LLIN	4638	320	14.5 (23.2)	0.74 (0.37–1.48)	0.3946	16/1432 (1.1, 0.7–1.8)	1.11 (0.52–2.38)	0.7941	0.22 (0.57)	1.00 (0.40–2.50)	0.9957
Outdoor	Pyr LLIN	4623	320	14.4 (24.1)	1 (Ref)		21/1753 (1.2, 0.7–2.1)	1 (Ref)		0.15 (0.34)	1 (Ref)	
	Pyr-PPF LLIN	4262	320	13.3 (18.8)	1.10 (0.57–2.10)	0.7870	21/1378 (1.5, 0.9–2.5)	1.06 (0.51–2.23)	0.8664	0.20 (0.57)	1.37 (0.57–3.30)	0.4815
	Pyr-CFP LLIN	3621	320	11.3 (19.3)	0.71 (0.37–1.36)	0.3017	11/1115 (0.9, 0.6–1.7)	0.70 (0.30–1.65)	0.4193	0.08 (0.24)	0.58 (0.19–1.74)	0.3320

*N* Number, *An* *Anopheles* vector (*An. gambiae* s.l. + *An. funestus* gr + *An. nili* gr), *SD* standard deviation, *DR* Density Ratio, *SR* Sporozoite Rate, *OR* Odd Ratio, *CI* Confidence Interval.

No reduction of the indoor SR was observed in either intervention arm [SR = 1.4%, OR 1.34 (95% 0.65–2.77), p = 0.4300 for the pyrethroid-pyriproxyfen LLIN arm, and SR = 1.1%, OR 1.11 (95% 0.52–2.38), p = 0.7941 for the pyrethroid-chlorfenapyr LLIN arm], compared to the standard pyrethroid-only LLIN arm (SR = 0.9%). The same was observed outdoors (Table 1).

Regarding the EIR, there was also no reduction observed indoors in either the pyrethroid-pyriproxyfen LLIN arm [EIR = 0.24 ib/p/n (infective bite/person/night), RR 1.10 (95% CI 0.44–2.72), p = 0.8380] and the pyrethroid-chlorfenapyr LLIN arm [EIR = 0.22 ib/p/n, RR 1.00 (95% CI 0.40–2.50), p = 0.9957], compared to the standard pyrethroid-only LLIN arm (EIR = 0.25 ib/p/n). A similar trend occurred outdoors (Table 1).

### Parous rate (PR) in *An. gambiae* s.l.

Overall, there was no strong evidence of a reduction in the parous rate in the two intervention arms relative to the standard pyrethroid-only LLIN arm, both indoors [PR = 76.9%, OR 0.78 (95% CI 0.49–1.25), p = 0.3063 in the pyrethroid-pyriproxyfen LLIN arm, and PR = 78.0%, OR 0.87 (95% CI 0.54–1.39), p = 0.5756 in the pyrethroid-chlorfenapyr LLIN arm, versus PR = 79.9% in the pyrethroid LLIN arm] and outdoors [PR = 75.5%, OR 0.58 (95% CI 0.35–0.98), p = 0.0416 in the pyrethroid-pyriproxyfen LLIN arm, and PR = 78.8%, OR 0.81 (95% CI 0.47–1.37), p = 0.4323 in the pyrethroid-chlorfenapyr LLIN arm, versus PR = 82.0% in the pyrethroid LLIN arm] (Table 2).

**Table 2:** Parous rate in *An. gambiae* s.l.

Locations	Arms	Total tested	N parous	% (95% CI)	OR (95% CI)	p-value
Indoor	Pyr LLIN	1369	1089	79.9 (76.0–83.9)	1 (Ref)	
	Pyr-PPF LLIN	1292	977	76.9 (70.6–83.3)	0.78 (0.49–1.25)	0.3063
	Pyr-CFP LLIN	1263	977	78.0 (71.8–84.2)	0.87 (0.54–1.39)	0.5756
Outdoor	Pyr LLIN	1217	989	82.0 (75.4–88.6)	1 (Ref)	
	Pyr-PPF LLIN	1358	1027	75.5 (70.3–80.7)	0.58 (0.35–0.98)	0.0416
	Pyr-CFP LLIN	1090	870	78.8 (74.2–83.5)	0.81 (0.47–1.37)	0.4323

*Pyr LLIN* standard pyrethroid-only LLIN, *Pyr-PPF LLIN* pyrethroid-pyriproxyfen LLIN, *Pyr-CFP LLIN* pyrethroid-chlorfenapyr LLIN, *OR* odds ratio, *CI* confidence interval.

### Impact of pyriproxyfen on the fertility rate (FR) of *An. gambiae* s.l.

In the pyrethroid-pyriproxyfen LLIN arm, the fertility rate (FR = 58.5%) of *An. gambiae* s.l. appeared to be reduced compared to the standard pyrethroid-only LLIN arm (FR = 65.4%), but this was not significant [OR 0.18 (95% CI 0.19–1.37); p = 0.1866].

No clear reduction in the FR was observed in the pyrethroid-chlorfenapyr LLIN arm [FR = 69.1%, OR 0.88 (95% CI 0.34–2.32); p = 0.8068], relative to the standard pyrethroid-only LLIN arm (FR = 65.4%) (Table 3).

**Table 3:** Fertility rate (FR) in *An. gambiae* s.l.

Arms	N dissected	N Fertile	Fertility rate (95% CI)	OR (95% CI)	p-value
Pyr LLIN	332	217	65.4 (50.8–77.5)	1 (Ref)	
Pyr-PPF LLIN	82	48	58.5 (41.4–73.8)	0.18 (0.19–1.37)	0.1866
Pyr-CFP LLIN	68	47	69.1 (45.1–85.9)	0.88 (0.34–2.32)	0.8068

*N* total number, *OR* odd ratio, *Pyr LLIN* standard pyrethroid-only LLIN, *Pyr-PPF LLIN* pyrethroid-pyriproxyfen LLIN, *Pyr-CFP LLIN* pyrethroid-chlorfenapyr LLIN, *OR* odds ratio, *CI* confidence interval.

### Allelic frequency of the L1014F *kdr* mutation in *An. coluzzii* and *An. gambiae* s.s.

Irrespective of location, the allelic frequencies of the L1014F *kdr* mutation were overall similar between the two intervention arms and the control arm for each molecular species (*An. coluzzii* and *An. gambiae* s.s.) (Table 4).

**Table 4:** Allelic frequency of the L1014F *kdr* mutation in *An. coluzzii* and *An. gambiae* s.s.

Locations	Molecular species	Arms	N of *An. gamb*	RR	RS	SS	F (L1014F Kdr)	95% CI	OR (95% CI)	p-value
Indoor	*Anopheles coluzzii*	Std LLIN	646	402	202	42	77.9^a^	75.6–80.1	1 (Ref)	–
		PPF LLIN	485	259	179	47	71.9^b^	69.0–74.7	0.73 (0.6–0.88)	0.0011
		CFP LLIN	431	247	157	27	75.5^a^	72.7–78.4	0.88 (0.71–1.08)	0.219
	*Anopheles gambiae s.s*	Std LLIN	207	180	21	6	92.0^a^	89.4–94.6	1 (Ref)	–
		PPF LLIN	230	204	19	7	92.8^a^	90.5–95.2	1.12 (0.68–1.85)	0.6562
		CFP LLIN	213	181	28	4	91.5^a^	88.9–94.2	0.94 (0.57–1.53)	0.7998
Outdoor	*Anopheles coluzzii*	Std LLIN	585	344	196	45	75.6^a^	73.1–78.0	1 (Ref)	–
		PPF LLIN	369	221	118	30	75.9^a^	72.8–79.0	1.02 (0.82–1.26)	0.8719
		CFP LLIN	340	197	124	19	76.2^a^	73–79.4	1.03 (0.81–1.32)	0.7854
	*Anopheles gambiae s.s*	Std LLIN	169	150	11	8	92.0^a^	89.1–94.9	1 (Ref)	–
		PPF LLIN	192	173	14	5	93.8^a^	91.3–96.2	1.3 (0.74–2.3)	0.3642
		CFP LLIN	164	140	16	8	90.2^a^	87.0–93.5	0.8 (0.46–1.39)	0.4354

*Pyr LLIN* standard pyrethroid-only LLIN, *Pyr-PPF LLIN* pyrethroid-pyriproxyfen LLIN, *Pyr-CFP LLIN* pyrethroid-chlorfenapyr LLIN, *OR* odds ratio, *CI* confidence interval, *N of An. gamb* Number of tested *An. gambiae* s.l., *F* allelic frequency; ^a,b,c,d^Values of F(L1014F *kdr*) with the same superscript in a given species within a same location are statistically similar.

## Discussion

Current LLIN procurement and deployment strategies are based on an anticipated operational longevity of three years. However, there is a growing body of evidence from the field that net bio-efficacy, physical durability and attrition rates can vary substantially between countries and populations^9^. As novel vector control interventions are developed, including dual active-ingredient (AI) LLINs, it is crucial to understand their impact throughout their lifespan, to develop best practices for their use in pragmatic insecticide resistance management schemes^18,19^. In this study, during the third year of community usage, pyrethroid-chlorfenapyr and pyrethroid-pyriproxyfen LLINs showed no evidence of a significant reduction of vector density, SR and EIR. Similarly, parous and fertility rates did not significantly decrease in the two intervention arms compared to the control one. These results align with the lack of epidemiological impact seen between arms in the third year^20^ and contrast with the results of the first two years of the trial, where pyrethroid-chlorfenapyr LLINs had a greater impact on epidemiological and entomological indicators than the standard pyrethroid-only LLINs. Moreover, no significant increase in the L1014F *kdr* frequency was induced by the two dual AI LLINs compared to the standard pyrethroid-only LLIN.

A contributing factor which may explain the reductions in efficacy of pyrethroid-chlorfenapyr LLINs may include decreases in community usage over time (from 83% at 9 months post-distribution to 52% at 36 months post-distribution)^20^. In addition, reduction in biological efficacy of the partner insecticide (Chlorfenapyr) may also explained the lack of efficacy observed on entomological outcomes in the third year of the RCT. Indeed, after 2 years of community usage of LLINs in the present trial, there was a 75% reduction of chlorfenapyr concentration (52 mgAI/sqm at 24 months versus 208 mgAI/sqm when new) in pyrethroid-chlorfenapyr LLINs^20^, and after 3 years of use nearly no chlorfenapyr were left in the same type of nets evaluated in another RCT conducted in Tanzania^10^. In an associated study conducted in experimental huts in Tanzania, the decline in chlorfenapyr concentration was associated with reduced vector mortality following exposure to pyrethroid-chlorfenapyr LLINs that had been deployed in the field for two years or longer^19^. Findings of a trial conducted in Papua New Guinea also showed that the decreased bio-efficacy of long-lasting insecticidal nets was followed by the loss of their effectiveness on malaria transmission^21^.

Study findings contrast with those from the RCT in Tanzania, where pyrethroid-chlorfenapyr LLINs continued to significantly reduce indoor vector density by 54% and EIR by 68% in the third year post-net distribution^10^, though the net usage in Tanzania (23%) was considerably lower compared to Benin (52%). Additionally, despite indications of a waning insecticidal effect of Chlorfenapyr in the nets after one year^19^, the Tanzanian RCT demonstrated ongoing effectiveness over a 3 years period. One possible explanation is the much (5–10 times) lower vector density in Tanzania compared to Benin. In Tanzania, the gradual build-up of the mosquito population would inherently take a longer period than in Benin. Therefore, the differences in entomological indicators between pyrethroid-chlorfenapyr LLINs and standard pyrethroid-only LLINs reported in the third year in Tanzania may be a carryover effect from the previous year rather than a true effect of the pyrethroid-chlorfenapyr LLINs.

In the study area, *An. gambiae* s.l. constituted the predominant *Anopheles* mosquitoes species, primarily composed of *An. coluzzii*, followed by *An. gambiae* s.s. This trend remained consistent with observation made before the net distribution and during the two first years post-intervention of the trial^11,22^, indicating that net deployment did not significantly change *Anopheles* species composition over time. This contrasts with findings in Burkina-Faso by Sanou et al.^23^, where the authors reported a significant shift in *Anopheles* species composition from *An. coluzzii* to *An. gambiae* s.s. as the most prevalent species following the introduction of LLINs.

In *An. gambiae* s.l., parous rates were similar between the pyrethroid-chlorfenapyr LLIN and standard pyrethroid-only LLIN arms in the present trial. The same observation was made over the two first years of the trial^22^. This might be due to highly variable confounding factors such as temperature and relative humidity that strongly influence parity^24^.

A three-minute exposure of mosquito tarsi to pyriproxyfen should suffice to inhibit their fertility^25^, therefore would expect a higher fertility rate in both the pyrethroid standard-only LLINs and the pyrethroid-chlorfenapyr LLIN arms as compared to the pyrethroid-pyriproxyfen LLIN arm, which did not occur in the present trial. This unexpected trend might be due to the decay of the pyriproxyfen incorporated into the nets as observed in the present study (90 mgAI/sqm at 24 months versus 289 mgAI/sqm when new: 69% reduction in pyriproxyfen concentration over 2 years).

The similar frequencies of the L1014F *kdr* mutation across study arms could be due to the mutation originally close to fixation in the whole study area due to successive mass LLIN distribution campaigns, and uncontrolled use of pyrethroids for agricultural purposes.

The present trial emphasizes the need to assess the long-term efficacy of any mosquito nets in the community, as this may vary over time, and from place to place. Indeed, findings of the present trial suggest that the two dual ai LLINs had an effective lifespan of two rather than three years, which corroborates previous findings by Gnanguenon et al.^26^. This needs to be taken into consideration by NMCPs, decision-makers, and LLINs procurement and distribution agencies which so far assume a three-year LLINs lifespan for any LLINs. Thus, to achieve a good efficacy of LLINs in the community, the replacement policy should be aligned with their operational lifespan. Moreover, manufacturers should develop LLINs with better materials, and their distribution should be followed by community sensitization on correct usage to avoid early appearance of holes, and repair of torn/holed nets.

## Conclusion

Findings from the present trial revealed that pyrethroid-chlorfenapyr LLINs were no longer better to standard nets against key entomological indicators of malaria transmission in their third year of community usage. There is a need for ongoing monitoring and evaluation of LLINs with dual active ingredients during their deployment to understand their effective lifespan in diverse epidemiological settings. This information is essential for malaria control programs to formulate appropriate net deployment strategies and guide manufacturers in developing longer-lasting nets to sustain control effort.

### Supplementary Information

Supplementary Figure S1.

## Data Availability

The datasets generated and/or analysed during the present study are available on reasonable request from the corresponding authors.
