# Supplementary material for: Efficacy of pyrethroid-pyriproxyfen and pyrethroid-chlorfenapyr nets on entomological indicators of malaria transmission: third year of a randomised controlled trial in Benin
Source: Sci Rep. 2024 Jun 5;14:12958. doi: 10.1038/s41598-024-63883-2 (PMC11153598; doi:10.1038/s41598-024-63883-2)
Supplement: Supplementary file 1 — Supplementary Figure S1. [file 41598_2024_63883_MOESM1_ESM.docx]

**Supplemental file**

**Figure S1.** Hourly mean indoor (N=16240) and outdoor (N=12331) density in *An. gambiae* s.l. in the study area
